# Supplementary material for: Clinical Utility of Comprehensive Genomic Profiling in Patients with Unresectable Hepatocellular Carcinoma
Source: Cancers (Basel). 2023 Jan 24;15(3):719. doi: 10.3390/cancers15030719 (PMC9913078; doi:10.3390/cancers15030719)
Supplement: Supplementary file 1 [file cancers-15-00719-s001.zip › cancers-2081680-supplementary.pdf]

Supplementary Materials: Clinical Utility of Comprehensive Genomic Profiling in Patients with Unresectable Hepatocellular Carcinoma

Shun Ishido, Kaoru Tsuchiya, Yoshihito Kano, Yutaka Yasui, Kenta Takaura, Naoki Uchihara, Keito Suzuki, Yuki Tanaka, Haruka Miyamoto, Michiko Yamada, Hiroaki Matsumoto, Tsubasa Nobusawa, Taisei Keitoku, Shohei Tanaka, Chiaki Maeyashiki, Nobuharu Tamaki, Yuka Takahashi, Hiroyuki Nakanishi, Urara Sakurai, Yasuhiro Asahina, Ryuichi Okamoto, Masayuki Kurosaki and Namiki Izumi

Table S1. Genomic results and clinical outcome in 9 patients.

**HCC Patients (*n* = 9)**	
Number of individuals with detectable alteration(s) (%)	9 (100%)
Number of individuals with ≥1 alterations (%)	9 (100%)
Median number of alterations (range)	4 (2–5)
Number of individuals who received molecular-targeted therapy (%)	1 (11.1%)
Median TMB* (range)	4 (0–20)
Number of individuals with TMB* ≥10 (%)	1 (11.1%)
Number of individuals with MSI** High (%)	0 (0%)
Number of individuals with ≥1 clinical trial options (%)	7 (77.8%)
Number of individuals who participated in clinical trial	0

Note: without VUS (variants of unknown significance), * means Tumor mutation burden, ** means Microsatellite Instability.

Table S2. The information of the clinical trial options and investigational candidate drugs.

**Patients number Age, Sex**	**Number of Clinical Trial Options**	**Investigational Candidate Drug**
① 20s, male	1	beta-catenin inhibitor
② 70s, female	3	beta-catenin inhibitor + Lenvatinib<br>FGFR inhibitor + PD-1 inhibitor<br>WNT inhibitor + PD-1 inhibitor
③ 30s, male	2	AKT inhibitor<br>MEK inhibitor
④ 70s, male	1	mTOR inhibitor
⑤ 50s, male	0	Nothing
⑥ 60s, male	7	PD-1 inhibitor<br>mTOR inhibitor<br>ATR inhibitor<br>beta-catenin inhibitor<br>MEK inhibitor
⑦ 60s, male	1	mTOR inhibitor
⑧ 60s, male	0	Nothing
⑨ 60s, male	1	ATR inhibitor

Commented [AAD1]: Please add an explanation in the table's footnote regarding the circled numbers.

Commented [s2R1]: Thanks for your comments. It means patients number, so we changed Table S2 as well as Table2,3.
